# Prostate Cancer Induced by Loss of Apc Is Restrained by TGFβ Signaling

**DOI:** 10.1371/journal.pone.0092800

**Published:** 2014-03-20

**Authors:** Glen A. Bjerke, Karolina Pietrzak, Tiffany A. Melhuish, Henry F. Frierson Jr., Bryce M. Paschal, David Wotton

**Affiliations:** 1 Department of Biochemistry and Molecular Genetics, and Center for Cell Signaling, University of Virginia, Charlottesville, Virginia, United States of America; 2 Department of Cytobiochemistry, University of Lodz, Lodz, Poland; 3 Department of Pathology, University of Virginia, Charlottesville, Virginia, United States of America; The Chinese University of Hong Kong, Hong Kong

## Abstract

Recent work with mouse models of prostate cancer (CaP) has shown that inactivation of TGFβ signaling in prostate epithelium can cooperate with deletion of the *Pten* tumor suppressor to drive locally aggressive cancer and metastatic disease. Here, we show that inactivating the TGFβ pathway by deleting the gene encoding the TGFβ type II receptor (*Tgfbr2*) in combination with a deletion of the *Apc* tumor suppressor gene specifically in mouse prostate epithelium, results in the rapid onset of invasive CaP. Micro-metastases were observed in the lymph nodes and lungs of a proportion of the double mutant mice, whereas no metastases were observed in *Apc* single mutant mice. Prostate-specific *Apc;Tgfbr2* mutants had a lower frequency of metastasis and survived significantly longer than *Pten;Tgfbr2* double mutants. However, all *Apc;Tgfbr2* mutants developed invasive cancer by 30 weeks of age, whereas invasive cancer was rarely observed in *Apc* single mutant animals, even by one year of age. Further comparison of the *Pten* and *Apc* models of CaP revealed additional differences, including adenosquamous carcinoma in the *Apc;Tgfbr2* mutants that was not seen in the *Pten* model, and a lack of robust induction of the TGFβ pathway in *Apc* null prostate. In addition to causing high-grade prostate intra-epithelial neoplasia (HGPIN), deletion of either *Pten* or *Apc* induced senescence in affected prostate ducts, and this restraint was overcome by loss of Tgfbr2. In summary, this work demonstrates that TGFβ signaling restrains the progression of CaP induced by different tumor suppressor mutations, suggesting that TGFβ signaling exerts a general tumor suppressive effect in prostate.

## Introduction

Prostate cancer (CaP) is one of the leading causes of cancer death in men, with the NCI predicting more than 230,000 cases and 29,000 deaths in the US in 2013 (www.cancer.gov/cancertopics/types/prostate). Several signaling pathways are frequently disrupted in human CaP, either by genetic alterations or by changes in expression of key components of the pathway [1]. Mutation or deletion of the *PTEN* tumor suppressor gene is found in more than 30% of primary human prostate cancers and more than 60% of CaP metastases [2]–[5]. Loss of *PTEN* lipid phosphatase activity activates the PI3-kinase signaling pathway [6], [7], including activation of the downstream Akt/PKB kinase, which is itself an oncogene [8]-[10]. Akt activation promotes cell survival by inhibiting apoptosis, and also regulates proliferation and cell size [11], [12].

Transforming growth factor (TGF) β ligands assemble a signaling complex of type I and type II receptors, in which the type II receptor phosphorylates and activates the type I [13]–[15]. The activated type I receptor then phosphorylates receptor Smad (R-Smad) proteins, which are the key transcription factors that mediate TGFβ family signaling. Smad2 and Smad3 are the primary R-Smads that respond to TGFβ signaling. Phosphorylated R-Smads bind Smad4 and accumulate in the nucleus where they regulate gene expression [16]. In many cell types, including epithelial cells, TGFβ signaling via Smad2/3 causes a G1 cell cycle arrest that prevents uncontrolled proliferation and plays a tumor suppressive role [17], [18]. The TGFβ signaling pathway is frequently disrupted by mutation or loss of expression of pathway components in human CaP [19]–[21]. Reduced expression of the TGFβ type I and type II receptors (encoded by the *TGFBR1* and *TGFBR2* genes) is associated with increased Gleason score and decreased survival, and reduced SMAD4 expression is also found in advanced human CaP [19], [22]–[24].

The *APC* (*Adenomatous Polyposis Coli*) gene encodes a tumor suppressor that is inactivated in a large proportion of human colorectal cancers [25]. Loss of APC function activates downstream components of the canonical WNT signaling pathway [26], [27]. APC functions as a scaffold that targets β-catenin for phosphorylation and subsequent proteasomal degradation. In the presence of WNT ligands, or a mutation in the APC gene, β-catenin is no longer degraded and can accumulate in both the cytosol and the nucleus, where it activates target gene expression primarily via interaction with the TCF/LEF DNA binding transcription factors [28]. Although the *APC* gene encodes a major tumor suppressor gene for colorectal cancer, and *APC* mutations have been found in some other cancers, inactivating mutations in the *APC* gene are rare in human CaP [29]. However, in the majority of cases of advanced human CaP β-catenin is found in the nucleus, suggesting that this pathway is frequently de-regulated. Other mechanisms for activation of this pathway have been identified in human CaP, including methylation of the *APC* gene [30], [31]. Activating mutations in β-catenin itself (encoded by *CTNNB1*), that prevent phosphorylation and targeting to the proteasome, have also been identified [32]. While de-regulation of this pathway appears to be quite frequent in human CaP, the importance of nuclear β-catenin in the initiation of human CaP and progression to castration resistant prostate cancer (CRPC) remains to be elucidated.

Recent work with genetically engineered mouse models has begun to shed light on the combinatorial effects of tumor suppressor mutations in prostate cancer progression. Prostate-specific deletions of the *Tgfbr2* and *Smad4* genes have both been tested in mice. Neither mutation alone is sufficient to initiate tumorigenesis, but when combined with a *Pten* deletion, inactivation of the TGFβ pathway results in very rapid progression to locally invasive and metastatic disease [33], [34]. Mouse models in which a stabilized β-catenin transgene was expressed in the prostate resulted in high-grade prostate intra-epithelial neoplasia (HGPIN) [35], [36]. Deletion of the *Apc* gene specifically in mouse prostate epithelium also results in HGPIN with high penetrance, but this rarely progresses to invasive cancer, and metastases were not found [37]. Here we show that deletion of both the *Tgfbr2* and *Apc* genes in mouse prostate epithelium results in rapid progression to invasive cancer, with metastases to lymph nodes and lungs in some cases. Additionally, we show that deletion of either *Apc* or *Pten* alone induces senescence in prostate ducts with HGPIN and that additional loss of the *Tgfbr2* gene overcomes this senescence checkpoint. In summary, this work suggests that TGFβ signaling limits progression from HGPIN to invasive prostate cancer, irrespective of the tumor initiating mutation. Thus TGFβ signaling plays a key tumor suppressive role in prostate epithelium.

## Results and Discussion

### Lethal prostate cancer in *Apc^r/r^;Tgfbr2^r/r^* mutants

**Figure 1:**
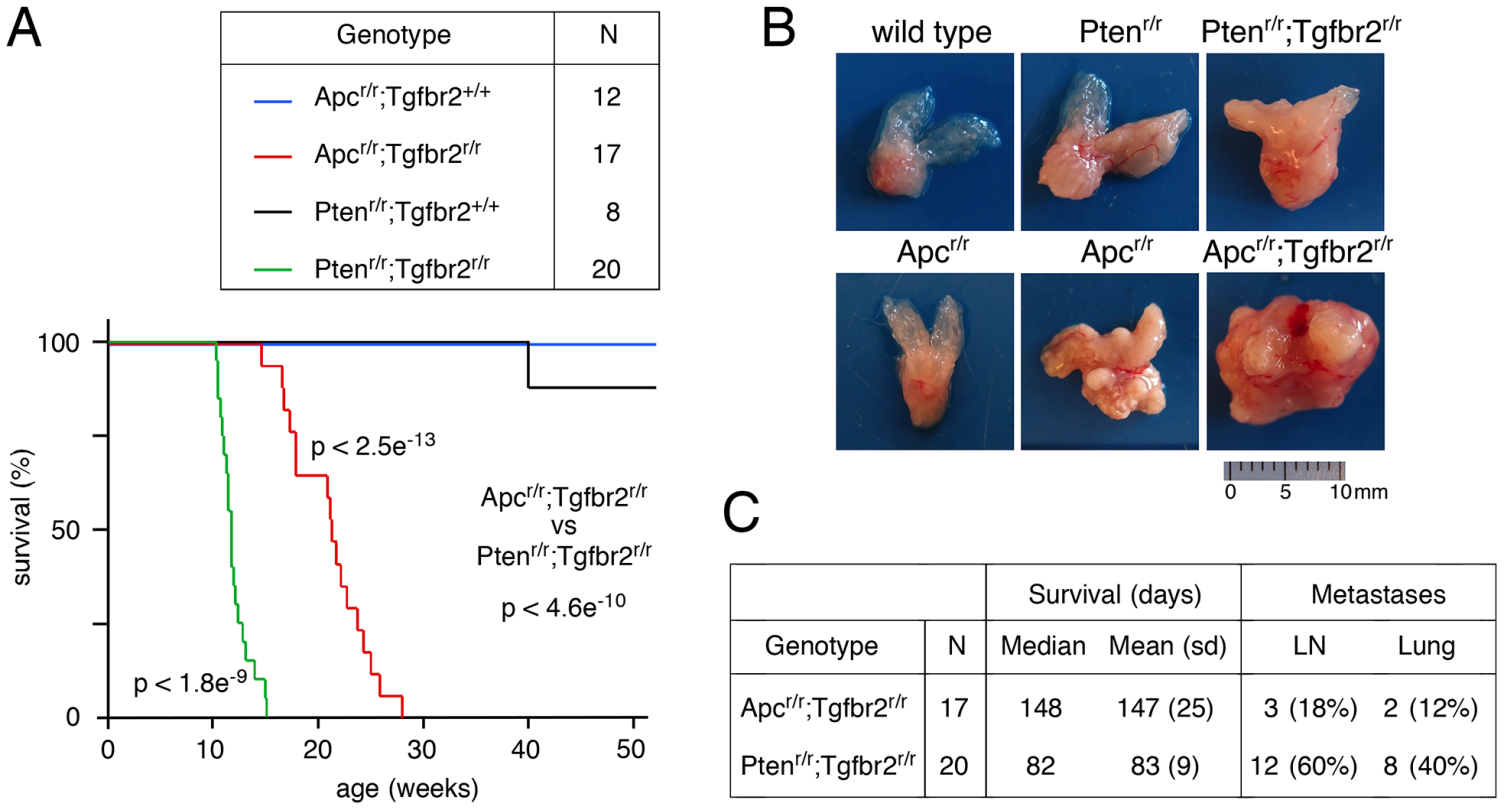
Deletion of *Tgfbr2* accelerates *Apc* null prostate cancer progression. (A) Kaplan-Meier survival curves are shown for each genotype as indicated. The number of animals for each is shown above. The p-values (log-rank test) are shown for *Apc^r/r^;Tgfbr2^r/r^* versus *Pten^r/r^;Tgfbr2^r/r^*, for *Apc^r/r^;Tgfbr2^r/r^* versus *Apc^r/r^*, and for *Pten^r/r^;Tgfbr2^r/r^* versus *Pten^r/r^*. (B) Gross anatomy of the prostate tumors is shown for each at the following ages: wild type (18 weeks), *Pten^r/r^* (18 weeks), *Pten^r/r^;Tgfbr2^r/r^* (13 weeks), *Apc^r/r^* (18 and 52 weeks), and *Apc^r/r^;Tgfbr2^r/r^* (15 weeks). (C) The survival data for each of the double nulls is shown, along with a summary of metastases to lumbar lymph nodes (LN) and lung. The proportion of mice with metastases is significantly different, comparing the *Pten^r/r^;Tgfbr2^r/r^* and *Apc^r/r^;Tgfbr2^r/r^* groups in C (p<0.01).

Homozygous deletion of *Apc* in mouse prostate epithelium results in HGPIN with squamous differentiation in all prostatic lobes, although it rarely progresses to locally invasive cancer, and metastases have not been detected [37]. TGFβ signaling restrains the progression of prostate cancer initiated by loss of the tumor suppressor Pten [33], [34]. To test whether TGFβ signaling plays a similar role in prostate when Apc is lost, we used mouse models to combine mutations in the *Tgfbr2* and *Apc* genes. Conditional, loxP-flanked alleles of both the *Apc* and *Tgfbr2* genes were combined with the *PbCre4* transgene, which is expressed only in prostate epithelium [38]. The recombined alleles, generated by prostate epithelium-specific expression of PbCre4, are referred to hereafter as *Apc^r/r^* and *Tgfbr2^r/r^*. As a first test of whether combining the *Apc* and *Tgfbr2* mutations promotes prostate cancer progression, we followed a cohort of *Apc^r/r^* and *Apc^r/r^;Tgfbr2^r/r^* mice to one year of age. Over this time course, none of the *Apc^r/r^* mice showed signs of distress, whereas all of the double mutants displayed a tumor burden that required euthanasia prior to 30 weeks of age (Figure 1A). For comparison, we also analyzed a number of *Pten^r/r^* and *Pten^r/r^;Tgfbr2^r/r^* mice over the same period. All but one of the *Pten* single mutants survived to one year, whereas none of the double mutants survived beyond 16 weeks, consistent with our previous analysis (Figure 1A and [33]). At 18 weeks of age, the prostates of *Apc* single null mutants were almost indistinguishable from the wild-type, whereas, prostatic enlargement was readily apparent in *Pten* nulls (Figure 1B). By 52 weeks, the *Apc* null prostate tumors were clearly evident, but were still much smaller than those in *Apc;Tgfbr2* double null mice at 16 weeks of age (Figure 1B). Thus, deletion of the *Tgfbr2* gene in the background of loss of either *Pten* or *Apc* had a highly significant effect on tumor growth, and reduced the median survival times to 82 days and 148 days respectively (Figure 1A and C). There was also a significant survival difference between the two double mutant combinations that is likely due to the specific pathways affected by loss of Pten and Apc.

We previously found that a relatively high proportion (∼ 66%) of *Pten^r/r^;Tgfbr2^r/r^* mice developed metastases to the lumbar lymph nodes or lung [33]. In the present study, micro-metastases were found in the lumbar lymph nodes of 18% of the *Apc^r/r^;Tgfbr2^r/r^* mice, and in the lungs of 12% of the *Apc^r/r^;Tgfbr2^r/r^* mice. Thus, metastases in the *Apc^r/r^;Tgfbr2^r/r^* mice were significantly less frequent (p<0.01) than in the *Pten^r/r^;Tgfbr2^r/r^* mutants examined here, and in our previous analysis (Figure 1C). We never observed metastases in *Apc^r/r^* single mutant mice, even by one year of age, and only one of fourteen *Apc^r/r^* mice analyzed at either 36 or 52 weeks of age showed signs of locally invasive cancer. These data clearly show that the combination of mutations in the *Apc* and *Tgfbr2* genes can accelerate tumor progression to invasive and metastatic disease over that seen with loss of *Apc* alone.

Inactivation of TGFβ signaling, either by deletion of *Tgfbr2* or *Smad4*, accelerates the progression of *Pten* null CaP, and *Tgfbr2* deletion has also been shown to cooperate with a constitutively active Akt transgene to drive invasive cancer [33], [34]. Expression of a dominant negative TGFβ type II receptor transgene in the prostate was able to increase the severity of tumors initiated by prostate specific expression of T antigen, and resulted in increased metasases [39]. In each case, progression to invasive and metastatic disease is accelerated by loss of TGFβ signaling, even when the initiating mutation does not on its own result in metastases, as seen here with the *Apc* mutants. In summary, the results of these mouse models of CaP together with the data presented here suggest that TGFβ signaling plays a major tumor suppressive role in CaP.

### Adenosquamous carcinoma in *Apc^r/r^;Tgfbr2^r/r^* mutant prostate

**Figure 2:**
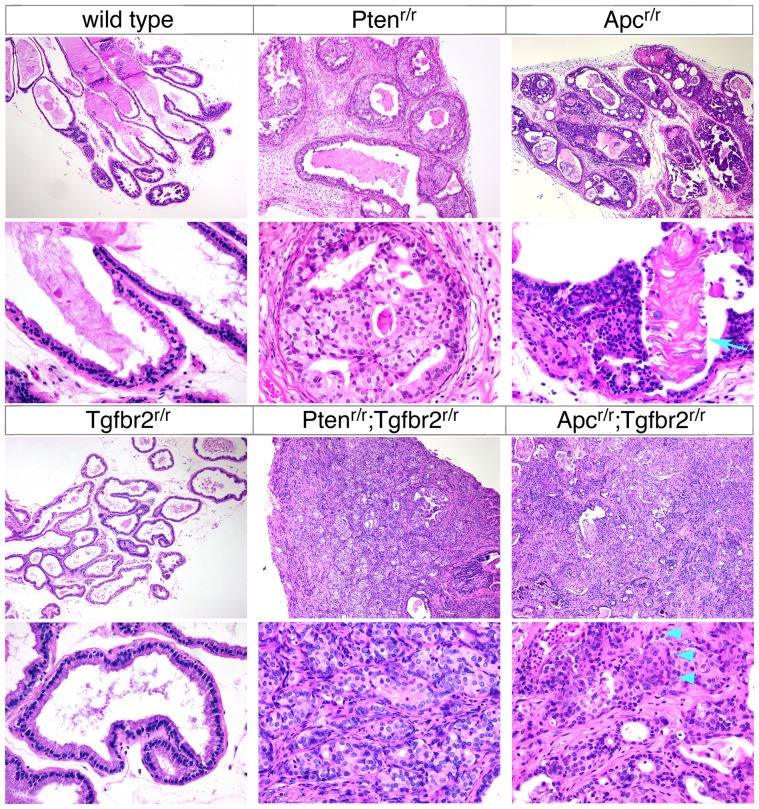
Invasive cancer in *Apc;Tgfbr2* null prostates. H&E stained sections are shown for the six indicated genotypes, taken from mice at the following ages. Arrow indicates keratin deposits in the *Apc^r/r^*, and arrowheads indicate an area of adenosquamous differentiation in the *Apc^r/r^;Tgfbr2^r/r^* prostate. Wild type: 21 weeks, *Pten^r/r^*: 22 weeks, *Apc^r/r^*: 36 weeks, *Tgfbr2^r/r^*: 70 weeks, *Pten^r/r^;Tgfbr2^r/r^*: 11 weeks, and *Apc^r/r^;Tgfbr2^r/r^*: 17 weeks. Images taken at 10× and 40× magnification are shown.

**Figure 3:**
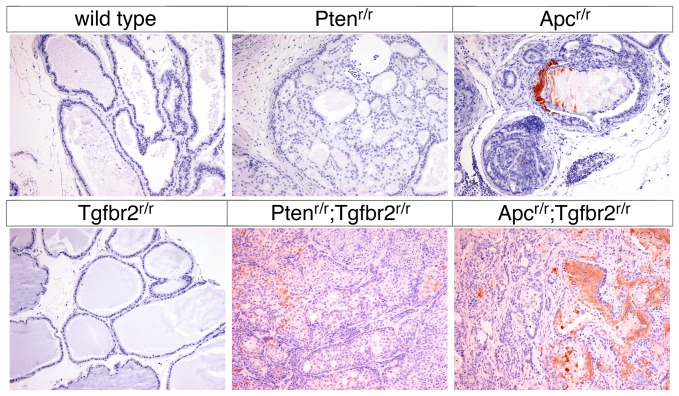
Keratin 10 staining in *Apc* null prostates. Prostates of the indicated genotypes were analyzed by IHC for Keratin 10, as a marker of squamous differentiation. Ages are as in Figure 2.

Given the differences in survival and in the gross appearance of the tumors from *Apc^r/r^;Tgfbr2^r/r^* and *Pten^r/r^;Tgfbr2^r/r^* mice we performed a more detailed comparison of these tumors, as well as of prostates from the single null mutants. As previously reported, histological analysis did not reveal any differences between the wild type and *Tgfbr2* null prostates (Figure 2 and [33]). Comparison of the *Pten* and *Apc* single mutants revealed HGPIN in prostates of both genotypes, with squamous differentiation in the *Apc* null that was not seen in the *Pten* null. Introduction of a *Tgfbr2* mutation resulted in progression to poorly differentiated adenocarcinoma (PDA) in all *Pten;Tgfbr2* double mutants. When combined with the *Apc* mutation, deletion of the *Tgfbr2* gene in prostate resulted in progression to invasive cancer, although the majority of *Apc^r/r^;Tgfbr2^r/r^* tumors retained the squamous phenotype seen in *Apc* single nulls (Figure 2). Note the prominent foci of keratin in the *Apc* null HGPIN (arrow, Figure 2) and adenosquamous differentiation indicated in the *Apc^r/r^;Tgfbr2^r/r^* tumor shown in Figure 2 (arrowheads). In addition to the histological appearance, increased expression of keratins 1 and 10 has been used as a marker of squamous differentiation in the *Apc* null prostate [40]. We, therefore, stained sections of wild type and mutant prostate for keratin 10. As shown in figure 3, strong staining for Krt10 was observed in cells underlying the prominent foci of keratin present in *Apc^r/r^* HGPIN, with little or no staining evident in wild type, *Tgfbr2* null or *Pten* null prostates. In the *Apc^r/r^;Tgfbr2^r/r^* tumors large regions of Krt10 positive cells were present throughout the tumor, whereas in the *Pten;Tgfbr2* double mutants only rare scattered Krt10 positive cells were seen (Figure 3).

**Figure 4:**
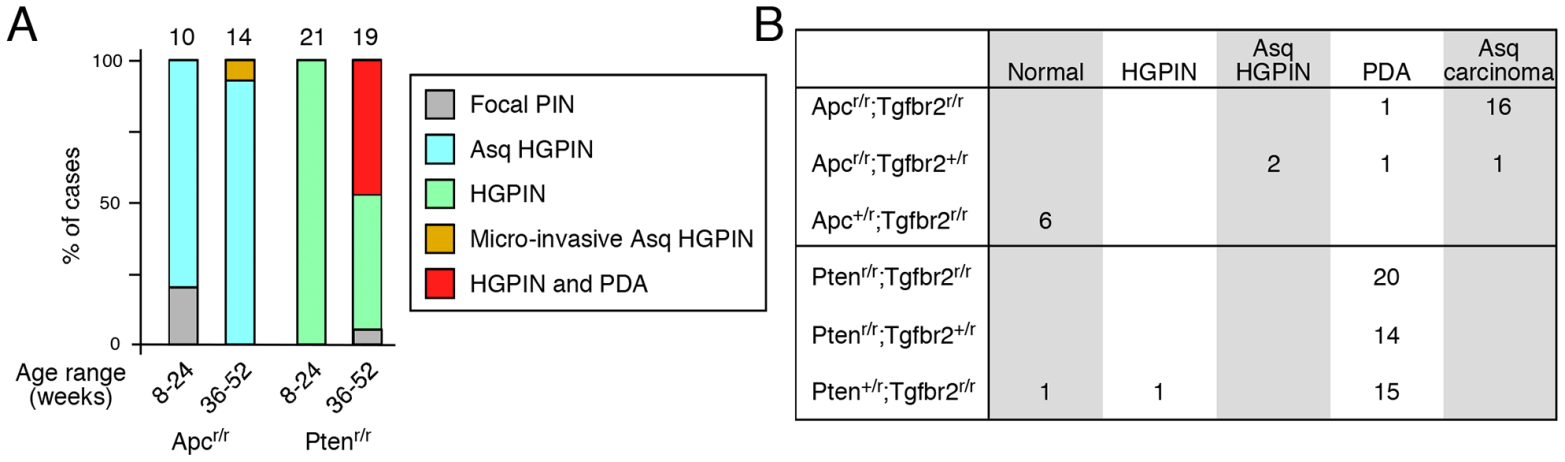
Summary of tumor phenotypes in mice with different combinations of *Apc*, *Pten* and *Tgfbr2* mutations. (A) The phenotypes of *Apc^r/r^* and *Pten^r/r^* mice are shown, grouped by two age ranges: 8 to 24 weeks and 36 to 52 weeks. All animals analyzed had some prostate tumor phenotype, which is classified as Focal PIN, HGPIN, or HGPIN with adenosquamous differentiation (Asq-HGPIN). Animals with micro-invasive cancer and Asq-HGPIN or a mixture of HGPIN and PDA are grouped separately. Numbers of animals analyzed are shown above each column, and the distribution of phenotypes is shown as a percentage. The proportion of animals with any signs of invasive cancer at 36–52 weeks is significantly different between the two genotypes (p<0.02). (B) The numbers of mice with each phenotype are shown for animals with different combinations of mutations in both *Apc* and *Tgfbr2* (above), or in both *Pten* and *Tgfbr2* (below). For each genotype the numbers of animals with each phenotype are shown. Normal – no tumor phenotype evident. HGPIN and adenosqamous HGPIN – extensive HGPIN without evidence of invasion. PDA and adenosqamous carcinoma – extensive locally invasive cancer. All mice were euthanized at more than one year of age, unless they had to be sacrificed for tumor burden at a younger age. Significantly more animals with invasive cancer were observed in the *Pten^r/r^;Tgfbr2^+/r^* and *Pten^+/r^;Tgfbr2^r/r^* groups than in the *Apc^r/r^;Tgfbr2^+/r^* and *Apc^+/r^;Tgfbr2^r/r^* groups (14/14 vs 2/4; p<0.05, and 15/17 vs 0/6; p<0.001). One of the *Apc^r/r^;Tgfbr2^+/r^* mice (scored as PDA) had only small invasive foci. All others had extensive local invasion if scored as PDA or Asq-carcinoma.

To better compare the frequencies of the phenotypes described above we compiled data on the predominant phenotypes in each genotype as scored in a blinded manner based on established mouse prostate phenotypes [41]–[43]. In *Apc^r/r^* mice most prostates analyzed at 8–24 weeks of age had HGPIN with squamous differentiation (adenosquamous HGPIN; Asq-HGPIN) and by 36–52 weeks all had this phenotype (Figure 4A). Only one of the *Apc* nulls developed regions of locally micro-invasive cancer (at 36 weeks), and this was significantly less frequent (p<0.02) than the incidence of invasive cancer in *Pten^r/r^* mice in the 36–52 week age range (Figure 4A). Comparison of the *Tgfbr2* compound mutants revealed that all *Pten^r/r^;Tgfbr2^r/r^* mice had PDA without squamous differentiation, whereas all but one of the *Apc^r/r^;Tgfbr2^r/r^* mice euthanized for tumor burden had extensive adenosquamous carcinoma (Figure 4B). We also compared phenotypes in mice with intermediate genotypes, in which one or other mutation was heterozygous (some of the *Pten^+/r^;Tgfbr2^r/r^* and *Pten^r/r^;Tgfbr2^+/r^* mice included in this phenotype summary were analyzed for survival in ref [33]). For this analysis, mice were analyzed when tumor burden became excessive, or when mice reached at least one year of age. This showed that the frequency of invasive cancer was significantly higher in *Pten^r/r^;Tgfbr2^+/r^* than in *Apc^r/r^;Tgfbr2^+/r^* mice (p<0.05), and in *Pten^+/r^;Tgfbr2^r/r^* than in *Apc^+/r^;Tgfbr2^r/r^* prostates (p<0.001), supporting the idea that loss of *Pten* results in a more aggressive phenotype than loss of *Apc* (Figure 4B).

In summary, this analysis suggests that the phenotypes observed are quite specific to each prostate cancer model, and that within each model there is little variation in the type of tumor. For all combinations tested the *Pten* mutation results in a more aggressive, invasive cancer than *Apc* deletion. Interestingly, none of the *Apc* heterozygotes developed tumors, even in the presence of a homozygous deletion of *Tgfbr2*, whereas the majority of *Pten* heterozygotes developed PDA if they were null for *Tgfbr2*. This is consistent with inactivation of the remaining *Pten* allele, which has been suggested to be a major route by which *Pten* heterozygous mouse prostates develop a phenotype [44]. TGFβ signaling appears to restrain the progression of HGPIN to both poorly differentiated adenocarcinoma and to adenosquamous carcinoma, with the difference in phenotype being dependent on the tumor initiating mutation. While nuclear accumulation of β-catenin is found in a high proportion of human CaP, there is no consensus as to how the APC/β-catenin pathway might be disrupted in human CaP. The squamous differentiation induced by loss of *Apc* is relatively rare in human CaP, although it is more common after androgen deprivation therapy [45]. The presence of adenosquamous carcinoma in *Apc;Tgfbr2* double nulls suggests that loss of TGFβ signaling does not prevent the squamous differentiation induced by loss of *Apc*. Similarly, the combination of a *Pten* mutation with a stabilized β-catenin transgene in prostate results in tumors with squamous differentiation, suggesting that this may be the dominant phenotype of early activation of β-catenin [40]. Perhaps the increased nuclear β-catenin seen in advanced human CaP is a later phenotype, or occurs to a lower level than seen with transgenic manipulation in mice, and thus cannot drive squamous differentiation.

### Increased stroma and invasive cancer with *Tgfbr2* deletion

**Figure 5:**
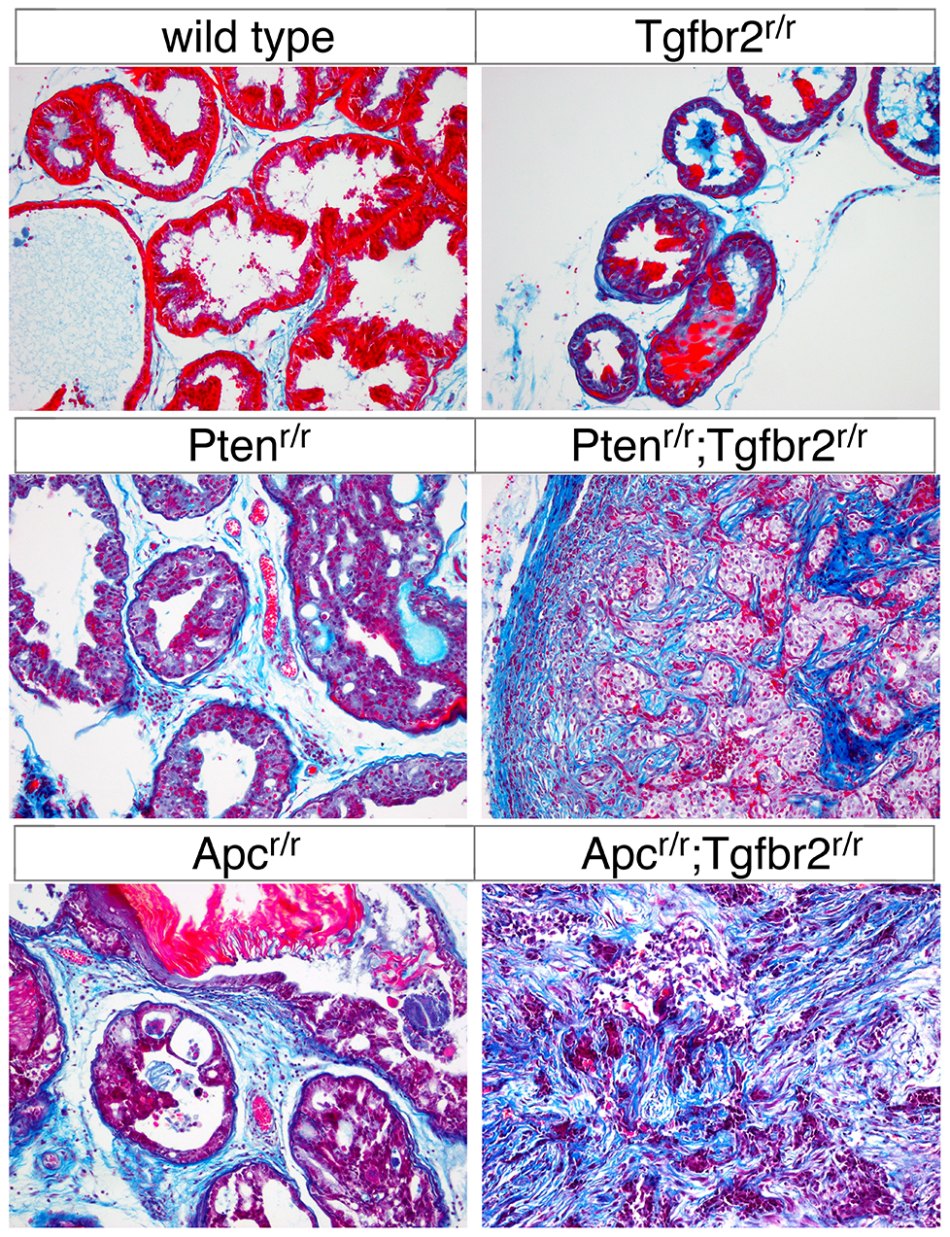
Increased stroma in *Apc* null prostates . Masson's Trichrome stained prostate sections are shown, from mice at the following ages. Wild type: 25 weeks, *Pten^r/r^*: 18 weeks, *Apc^r/r^*: 36 weeks, *Tgfbr2^r/r^*: 18 weeks, *Pten^r/r^;Tgfbr2^r/r^*: 12 weeks, and *Apc^r/r^;Tgfbr2^r/r^*: 17 weeks.

Examination of sections stained with H&E revealed evidence of locally invasive cancer, with increased stroma in both *Apc^r/r^;Tgfbr2^r/r^* and *Pten^r/r^;Tgfbr2^r/r^* prostates. To visualize expansion of the stroma, we stained sections with Masson's Trichrome. In wild type and *Tgfbr2* null prostates, the duct structure was similar, and there was minimal fibrous tissue between prostatic ducts. In areas of HGPIN in *Pten^r/r^* animals there were focal areas of increased fibrosis and in the *Apc* null significant fibrosis was seen between ducts with HGPIN (Figure 5). In areas of invasive cancer in both the *Apc^r/r^;Tgfbr2^r/r^* and *Pten^r/r^;Tgfbr2^r/r^* prostates, increased fibrosis was present, and this was particularly evident in the *Apc^r/r^;Tgfbr2^r/r^* mice (Figure 5).

**Figure 6:**
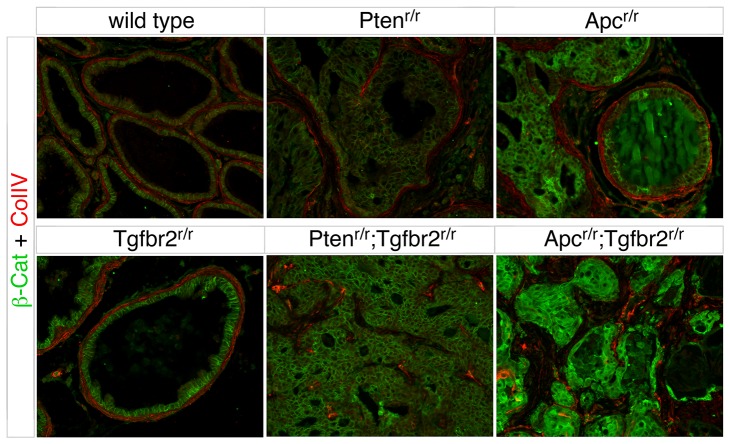
Basement membrane breakdown in double null prostates. Indirect immunofluorescence is shown for β-catenin (green) and Collagen IV (red) on sections of prostate from mice of the indicated genotypes. Wild type: 21 weeks, *Tgfbr2^r/r^*: 44 weeks, *Pten^r/r^*: 21 weeks, *Pten^r/r^;Tgfbr2^r/r^*: 11 weeks, *Apc^r/r^*: 36 weeks, and *Apc^r/r^;Tgfbr2^r/r^*: 24 weeks old.

**Figure 7:**
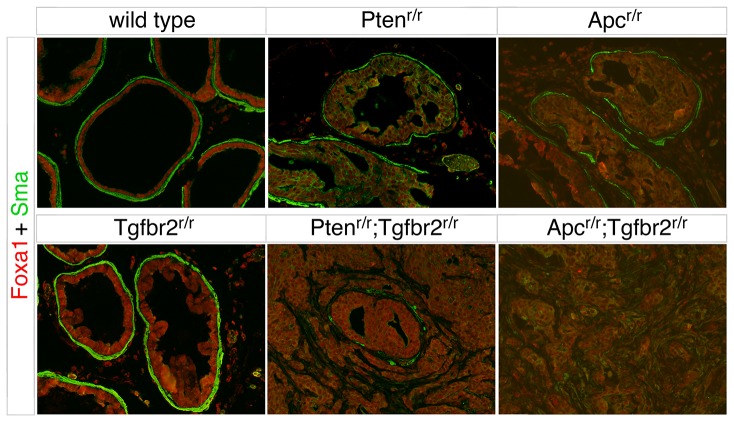
Disruption of stromal integrity in double null prostates. FoxA1 (red) and Sma (green) staining are shown by indirect immunofluorescence on sections of prostate from mice of the indicated genotypes. Ages of the mice are as in Figure 6.

We next examined the breakdown of duct structure in each of the two prostate cancer models. Immunostaining for Collagen IV, as a marker for basement membrane integrity, demonstrated that the basement membrane was intact in both wild type and *Tgfbr2* null prostates. In the *Apc* and *Pten* mutants (at 36 and 21 weeks of age respectively), there was minimal basement membrane breakdown around ducts with HGPIN (Figure 6). The ages of these mice are well beyond the median survival times of 21 and 12 weeks for each of the double mutants. In contrast, examination of double mutant animals that were euthanized due to tumor burden showed complete breakdown of the basement membrane in areas of invasive cancer (Figure 6). To examine the invasive phenotype, we stained sections for Foxa1, as an epithelial marker, and for Sma to identify the stromal cells. In both the wild type and *Tgfbr2* null, prostatic ducts were fully enclosed by Sma positive stromal cells. Although there was clear evidence of HGPIN in the *Apc* and *Pten* null prostates, in most cases the ducts were still surrounded by Sma-positive stroma at ages significantly greater than the median survival times of the double mutants (Figure 7). In contrast, in *Apc^r/r^;Tgfbr2^r/r^* and *Pten^r/r^;Tgfbr2^r/r^* animals, the distinct separation between stroma and ducts has clearly broken down, as duct structure is no longer evident upon transition to invasive cancer (Figure 7). Taken together, these analyses suggest that while there are some differences in the histologic type of cancer initiated by loss of Pten or Apc, the additional inactivation of the TGFβ pathway causes rapid progression to locally invasive cancer.

**Figure 8:**
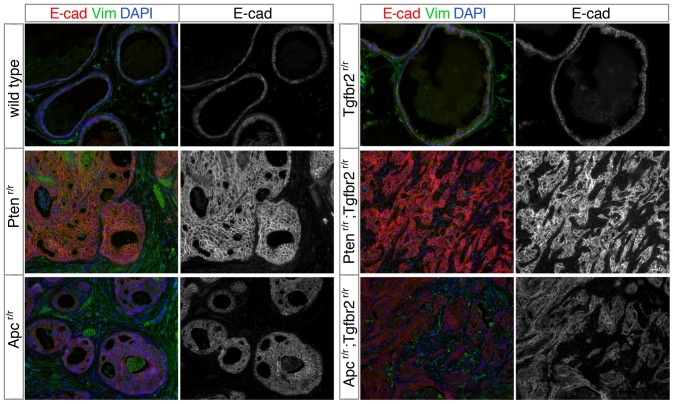
Expression of vimentin and E-cadherin in prostate. Prostates of the indicated genotypes, selected to be representative of the most common phenotype of each, were analyzed by indirect immunofluorescence for E-cadherin (red) and Vimentin (green). Ages are as follows, wild type: 21 weeks, *Pten^r/r^*: 45 weeks, *Apc^r/r^*: 52 weeks, *Tgfbr2^r/r^*: 70 weeks, *Pten^r/r^;Tgfbr2^r/r^*: 12 weeks, and *Apc^r/r^;Tgfbr2^r/r^*: 23 weeks.

To test for evidence of epithelial to mesenchymal transition (EMT) we examined expression of E-cadherin and vimentin. TGFβ signaling is a well known driver of EMT and increased TGFβ signaling frequently results in increased vimentin expression and a decrease in E-cadherin expression, which can contribute to the breakdown of epithelial cell junctions and an invasive phenotype [46]. As shown in Figure 8, E-cadherin is robustly expressed and present at the cell periphery in the majority of epithelial cells in the *Apc* and *Pten* single mutants. E-cadherin expression still appears largely normal in the *Pten^r/r^;Tgfbr2^r/r^* mice, despite the clear breakdown of duct structure. Some evidence of E-cadherin de-localization from the cell membrane was seen in the *Apc^r/r^;Tgfbr2^r/r^* prostate, together with a reduction in overall signal compared to the *Apc^r/r^*. However, even in the two double mutant prostates, epithelial cell junctions appear to be largely intact with clear E-cadherin staining (Figure 8). Vimentin staining revealed little change in in the epithelial cells in any of the mutants, although there was some increased vimentin expression in the stroma of *Apc* and *Pten* single null and *Apc;Tgfbr2* double null prostates (Figure 8). These data suggest that although the tumors in *Pten;Tgfbr2* and *Apc;Tgfbr2* double nulls are invasive and can metastasize, this is not accompanied by a large scale EMT phenotype. Given the role of TGFβ signaling in driving EMT, it might be expected that the double null tumors would not have down-regulated E-cadherin and up-regulated vimentin, as they have lost a key component of the TGFβ signal transduction pathway. These results do, however, leave open the question of how these tumors become metastatic. One possibility is that rare epithelial cells within the tumor have undergone EMT, presumably driven by a signal other than TGFβ. Another intriguing possibility is that the double null epithelial cells undergo some form of collective invasion, in which small groups of epithelial cells become invasive and motile, while maintaining their cell junctions. This type of invasion has been attracting more interest as a potential driver of metastasis [47], [48], and it will now be of interest to examine how the tumors examined here become invasive and metastatic.

### *Apc* deletion does not activate TGFβ signaling in prostate

**Figure 9:**
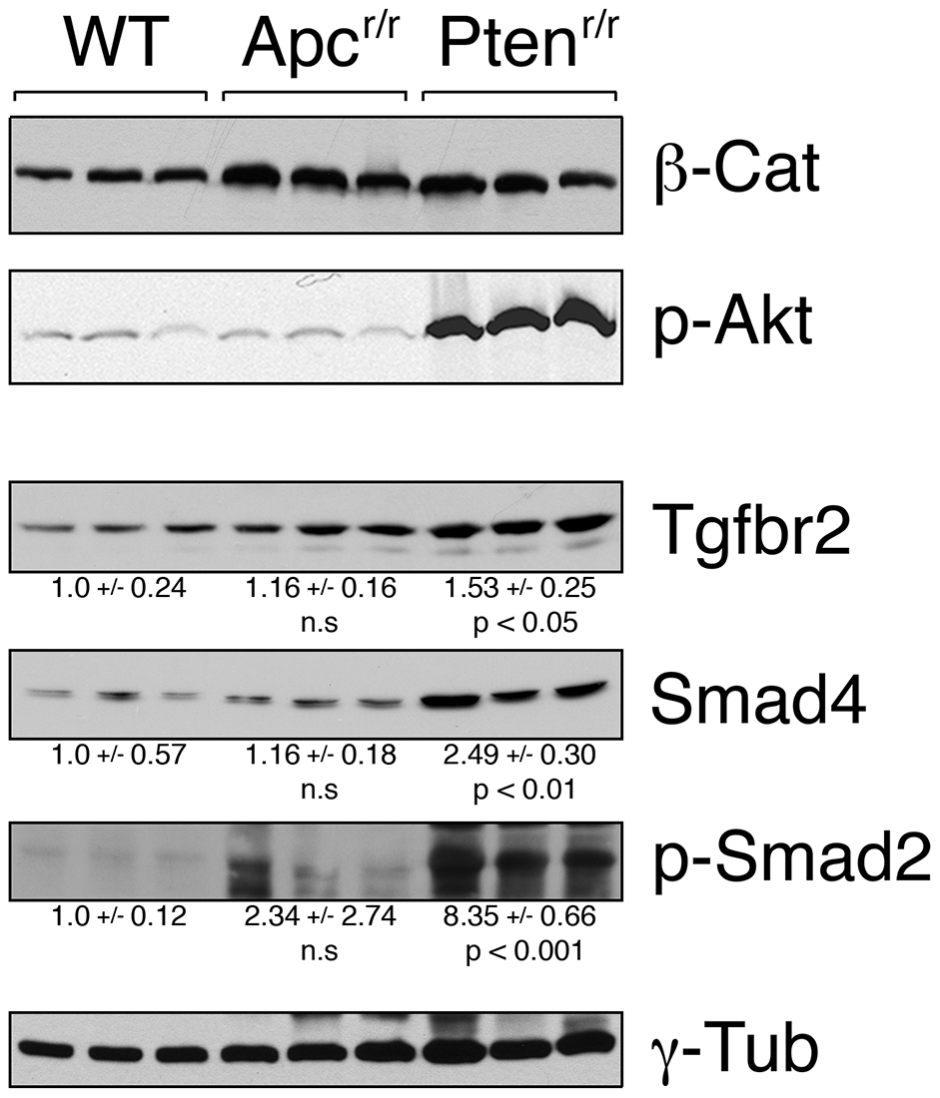
Analysis of the TGFβ pathway in *Apc* and *Pten* null prostate. Western blots are shown of lysates from the ventral prostates of 3 wild type, 3 *Apc^r/r^* and 3 *Pten^r/r^* mice as indicated. Lysates were analyzed by western blotting for β-catenin (β-cat), Akt1 phosphorylated on serine 473 (p-Akt), Tgfbr2, Smad4, and Smad2 phosphorylated on serines 465 and 467 (p-Smad2). Blotting for γ-tubulin is shown below as a loading control. Relative quantification (normalized to γ-tubulin) is shown (mean +/- s.d.) for the Tgfbr2, Smad4 and pSmad2 blots, together with p values for the comparison of *Pten* and *Apc* mutants to wild type.

Deletion of *Pten* in mouse prostate initiates tumorigenesis and induces the activity of the TGFβ pathway [33], [34]. Similarly, expression of a constitutively active *AKT1* transgene in prostate epithelium increases TGFβ signaling [33], suggesting that Akt activation, which occurs downstream of Pten loss, is sufficient to activate this pathway. Given that deletion of the *Tgfbr2* gene allowed for progression from HGPIN to invasive cancer in the *Apc* null prostate, we examined whether the TGFβ pathway was induced in this model. Deletion of either *Apc* or *Pten* resulted in little change in overall β-catenin levels, whereas phospho-Akt levels were increased specifically in *Pten* null prostates (Figure 9). To test whether the TGFβ pathway was affected by *Apc* deletion, we first analyzed levels of the TGFβ type II receptor and the intracellular mediator, Smad4. While both were significantly increased in the *Pten* null, there was no significant increase in either Smad4 or Tgfbr2 levels in *Apc* mutant prostates compared to those from wild type mice (Figure 9). As an indication of pathway activation, we next analyzed levels of Smad2 phosphorylated at the carboxyl-terminal serines that are a substrate for type I TGFβ receptors. Phospho-Smad2 was significantly increased in the *Pten* null but not in the *Apc* null prostates, indicating that pathway activation occurs with loss of Pten, but not with loss of Apc. The induction of TGFβ signaling by *Pten* deletion could be driven by signal transduction events downstream of Akt activation, or could be a consequence of the type of differentiation in this model – poor glandular differentiation in the *Pten* null rather than the squamous differentiation seen with *Apc* deletion. However, the clear cooperative effects of *Apc* and *Tgfbr2* deletion suggest that even the low level of basal TGFβ signaling present in the *Apc* mutant tumors is important for restraining cancer progression to locally invasive and metastatic disease.

### Loss of TGFβ signaling overcomes senescence

**Figure 10:**
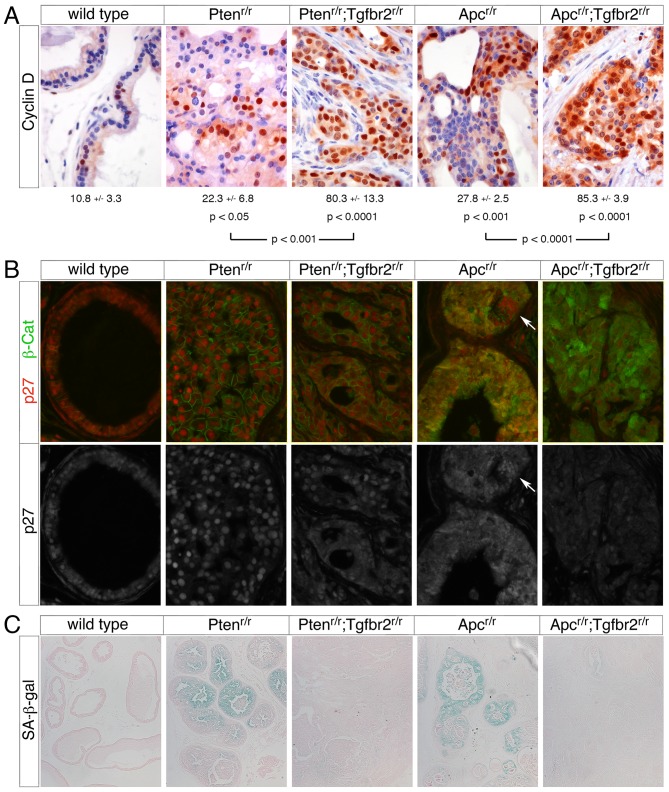
Induction of senescence is overcome by loss of Tgfbr2. (A) Cyclin D staining is shown by IHC (ages from left to right: 21, 22, 11, 36 and 17 weeks). Quantification is shown below (mean +/- s.d.) from four animals per genotype, except for *Apc^r/r^*, for which three mice were analyzed. The p values are shown (two-tailed Student's T test) for comparison of each genotype to wild type (above) and for comparison of each double to the relevant single mutant (below). (B) p27 and β-catenin expression was analyzed by indirect immunofluorescence. A merged image showing p27 in red and β-catenin in green is shown, together with a monochrome image of p27 alone (below). Arrows indicate a focus of cells with low β-catenin expression and high nuclear p27. Ages from left to right: 21, 43, 12, 36 and 18 weeks. (C) Senescence-associated β-galactosidase (SA-β-G) staining is shown (bottom row, ages from left to right: 19, 19, 10, 51 and 16 weeks) in sections of prostates from the indicated genotypes. Prostates were removed and ventral lobes stained overnight for SA-β-G, followed by sectioning and eosin counterstaining, prior to imaging.

To examine the effects of the *Tgfbr2* mutation on proliferation in *Pten* and *Apc* null tumors we examined expression of Cyclin D, increased levels of which correlate with advanced human prostate cancer [34]. As shown in Figure 10A, the number of epithelial cells with high levels of nuclear Cyclin D increased significantly in HGPIN in both the *Pten* and *Apc* single mutant prostates, with further significant increases in invasive cancer in each double mutant compared to the corresponding single mutant. We next examined expression of the CDK inhibitor, p27 (encoded by *Cdkn1b*) by immunofluorescence microscopy. For this analysis we co-stained for β-catenin to identify cells in which the *Apc* mutant phenotype was strongest. Expression of p27 increased in the *Pten* null and this increase was less pronounced in areas of invasive cancer in the *Pten^r/r^;Tgfbr2^r/r^* animals (Figure 10B). This observation suggests that the transition to invasive cancer is concomitant with decreased p27 expression, consistent with our previous analysis [33]. In the *Apc* mutant, we observed a dramatic change in the localization of β-catenin from the cell periphery to a more diffuse expression pattern throughout the cell. This change in β-catenin expression was accompanied by a redistribution of p27 from the nucleus, as seen in the wild type prostate, to a diffuse signal throughout the cell (Figure 10B). The change in p27 localization appeared to correlate with altered β-catenin expression, as small foci of cells in which β-catenin was still present at lower levels and only at the cell membrane retained nuclear p27 (Figure 10B arrow). Interestingly, in the *Apc^r/r^;Tgfbr2^r/r^* mutants p27 levels decreased relative to those seen in the *Apc* single mutant, suggesting that loss of Tgfbr2 has similar effects on p27 in both models (Figure 10B). These data are consistent with loss of TGFβ signaling resulting in increased proliferation in both models of prostate cancer, although the effects of *Pten* and *Apc* deletions appear to be different with respect to p27 expression, and it is possible that the p27 re-localization in the *Apc* null prostate represents a first step in its inactivation. Phosphorylation of p27 at threonine 157 by AKT1 down-regulates human p27, but this phosphorylation site is not conserved in the mouse protein [49]. Additionally, phosphorylation of p27 on other sites that are conserved between mouse and human, results in redistribution to the cytoplasm and in some cases subsequent proteasomal degradation [50]. However, we do not know if the effect of *Apc* deletion on p27 localization is due to phosphorylation of p27 itself.

A senescent phenotype can be induced in tumors either by activation of an oncogene, or by inactivation of a tumor suppressor gene [51]. Constitutive Akt activation in prostate epithelium induces cellular features of senescence, including SA-β-Gal (senescence-associated β-galactosidase) activity and increased p27 expression [52]. To test whether deletion of *Pten* and *Apc* induced senescence we analyzed the ventral prostates from different genotype mice for SA-β-Gal. Regions of HGPIN in both the *Apc* and *Pten* null prostates were positive for SA-β-Gal, whereas no staining was seen in the wild type prostate (Figure 10C). Areas of invasive cancer in the two double mutants were devoid of SA-β-Gal staining, although isolated regions of HGPIN in the double mutants did still retain some SA-β-Gal signal, suggesting that the transition from HGPIN to an invasive phenotype is associated with overcoming senescence (Figure 10C). These data suggest that deletion of either *Apc* or *Pten* in prostate epithelium initiates tumorigenesis, and also induces a senescent phenotype that can be overcome by deletion of the *Tgfbr2* gene, which allows rapid progression to invasive cancer.

Although *Apc* deletion in prostate has been shown to generate HGPIN with only rare progression to locally invasive cancer [37], to our knowledge this is the first study to examine the combination of *Apc* deletion with another mutation in prostate epithelium. Expression of stabilized β-catenin in prostate epithelium was able to cooperate with *Pten* deletion, or with expression of an activated *Ras* transgene to accelerate the onset of locally invasive cancer [35], [40]. However, either *Pten* deletion or *Ras* activation alone are sufficient to cause invasive CaP, albeit with slower kinetics than when combined with stabilized β-catenin. While stabilized β-catenin was able to accelerate the *Pten* null phenotype, deletion of β-catenin did not slow the progression of *Pten* null tumors to HGPIN, suggesting that it is not required for the early stages of tumorigenesis in this model [40]. We found that inactivation of TGFβ signaling results in a dramatic acceleration of tumor progression initiated by *Apc* deletion. Despite the lack of an abnormal phenotype seen with loss of TGFβ signaling in prostate epithelium, the combination of inactivating TGFβ pathway mutations with either *Pten* or *Apc* deletion results in highly aggressive mouse models of CaP. Thus, TGFβ signaling might restrain a relatively early step in the progression of these tumors, although it does not appear to affect tumor initiation.

It is possible that loss of TGFβ signaling contributes to the transition from androgen-sensitive to CRPC. Androgen deprivation therapy is one of the major treatments for biologically significant human CaP, but tumors virtually always return and in general are more aggressive after becoming castration resistant. Previous work suggests that early castration of prostate-specific *Apc* null mice slows the progression of tumors in this model [37]. By 32 weeks of age regions of hyperplasia and metaplasia were still evident following castration at six weeks, whereas intact animals at this age display adenosqamous HGPIN. We previously showed that the *Pten;Tgfbr2* null tumors were resistant to castration, suggesting that *Tgfbr2* deletion speeds the progression to CRPC. However, in this model it is difficult to separate out the effects of *Pten* deletion from *Tgfbr2* deletion, since HGPIN in *Pten* null mouse prostate has a limited response to castration. Therefore, the *Apc* model might present an opportunity to test whether inactivation of TGFβ signaling contributes to progression to CRPC.

In summary, we show that loss of TGFβ signaling in mouse prostate epithelium cooperates with loss of either the *Apc* or *Pten* tumor suppressor genes to drive invasive CaP, despite clear differences in the pathways activated and the tumor phenotypes. Loss of TGFβ overcomes a restraint on tumor progression resulting in rapid onset of invasive and metastatic disease, further supporting a major tumor suppressive role for TGFβ signaling in prostate.

## Materials and Methods

### Ethics statement

All animal procedures were approved by the Animal Care and Use Committee of the University of Virginia, which is fully accredited by the AAALAC.

### Mice and DNA analysis

The *loxP* flanked *Pten*, *Apc* and *Tgfbr2* alleles and the *Pb-Cre4* allele have been described previously [38], [53]–[55]. *Tgfbr2* and *Apc* mice, and the *Pb-Cre4* transgenics were obtained from the NCI mouse repository. Conditional loxP flanked alleles each contain loxP flanked exons, which when recombined result in null alleles, and are referred to here as ‘r’ for recombined (null), or ‘f’ for the conditional loxP flanked (equivalent to wild type). All mouse lines were maintained on a mixed C57BL/6J x FVB strain background, as previously described [33]. Genomic DNA for PCR genotype analysis was purified from ear punch, at post-natal day 21 (P21), by HotShot [56], and genotypes were determined by PCR.

### Histology, IHC and IF

Prostates were fixed in zinc-formalin, paraffin-embedded and stained with Hematoxylin and Eosin (H&E) or with Masson's Trichrome by standard techniques. Immunohistochemistry (IHC) and immunofluorescence (IF) analyses were performed as previously described [57]–[60]. Whole-mount prostate images were taken with a Leica MZ16 stereomicroscope and QImaging 5.0 RTV digital camera. IF images were captured on an Olympus BX51 microscope and DP70 digital camera, or on a Nikon Eclipse NI-U and captured with a DS-QI1 camera with NIS Elements software. Images were manipulated in Adobe Photoshop CS6. Antibodies for IF and IHC were against: Smad4 (Millipore 04-1033) phospho-Akt (Cell Signaling 9277), Cyclin D (Santa Cruz sc-753), p27 (BD Transduction Labs 610242), Foxa1 (Everest Biotech EB05999), Sma (Epitomics E184), β-catenin (BD Transduction Labs 610153), Collagen IV (AbD Serotech 2150-1470), E-cadherin (Cell Signaling 3195), vimentin (Abcam Ab20346), Krt10 (Covance PRB-159P).

### Senescence-associated β-galactosidase (SA-β-G) staining

Ventral prostates were fixed for 20 minutes in PBS + 4% paraformaldehyde, then washed 3 times in detergent wash (100 mM phosphate buffer pH 7.4, 2 mM MgCl_2_, 0.01% sodium deoxycholate, 0.02% NP-40). Whole lobes were stained overnight at 37°C in detergent wash with 5 mM potassium ferricyanide, 5 mM potassium ferrocyanide, 1 mg/ml X-gal and 20 mM Tris pH 7.5, then post-fixed in 4% paraformaldehyde overnight at 4°C. They were then dehydrated through ethanol (70%, 80%, 90%, 95%, 100% × 2), washed twice in xylene for 30 minutes, once in 50% xylene/50% wax for 1 hour, once in liquid wax for 1 hour, and then incubated overnight in liquid wax. Blocks were sectioned and counterstained with eosin. Images were captured on a Nikon Eclipse NI-U and a DS-RI1 camera.

### Western blotting

Proteins were separated by SDS-PAGE, transferred to Immobilon-P (Millipore) and proteins were visualized using SuperSignal West Pico ECL (Pierce). Primary antibodies were against β-catenin (BD Transduction Labs 610153), phospho-Akt (Cell Signaling 9277), phospho-Smad2 (Millipore AB3849), Smad4 (Millipore 04-1033), Tgfbr2 (Novus NBP1-19434) and γ-tubulin (Sigma T6557). Blots were quantified by densitometry using Image J.

### Statistics

Comparisons of proportions (Figures 1C, 4A and 4B) were performed using an N-1 Chi squared analysis of 2 × 2 contingency tables. Survival data (Figure 1A) was compared by log-rank test (http://bioinf.wehi.edu.au/software/russell/logrank/). Significantly increased protein expression (Figure 9) was tested for using a one-tailed Student's T test. Differences in Cyclin D expression (Figure 10A) were tested using a two-tailed Student's T test.
